# Moving Beyond Health to Flourishing: The Effects of Yoga Teacher Training

**DOI:** 10.1100/tsw.2010.87

**Published:** 2010-05-04

**Authors:** L. A. Conboy, A. Wilson, T. Braun

**Affiliations:** ^1^ Osher Research Center, Harvard Medical School, Boston, MA, USA; ^2^ Kripalu Center for Yoga and Health, Stockbridge, MA, USA

**Keywords:** measurement development, yoga, quality of life

## Abstract

Research in the medical and psychological fields has primarily followed a “disease-focused” approach to health. Although there is growing research on the components and outcomes of well-being, very few studies have focused on traditional practices that can be used as interventions to encourage human flourishing. The current study was developed to address this research gap. We suggest one effective method of increasing psychological well-being, the practice of yoga, an age-old practice that has been said to produce physical and psychological health. In this observational study, we examined associations with participation in a 4-week yoga teacher training resident program. Measurement instruments were chosen to capture changes in psychosocial health and human flourishing. Measurements were taken before the start of the program, immediately after the program, and 3 months postprogram. As expected, in this healthy population, the human flourishing scales showed more change than the psychosocial health scales. For example, in this healthy sample, there were no significant changes in perceived social support, quality of life, or self-efficacy from baseline to the 3-month follow-up. However, optimism, a positive psychology research measure, improved from baseline to follow-up. The mindfulness subscales of observation, awareness, and nonreactivity all improved following the training, suggesting that one benefit of yoga practice is a more refined ability to attend to one's inner experience. This study adds to the growing literature focusing on interventions that move beyond relieving pathology to those that produce optimal functioning and human thriving.

## INTRODUCTION

Research in the medical and psychological fields has primarily followed a "disease-focused" approach to health[1]. Behavior change research has traditionally focused on eliminating negative health behaviors and solidifying behaviors that have a direct known biological effect (such as dietary changes). In the field of psychology, since World War II, psychological research has focused primarily on curing mental disorders, rather than researching optimal states of psychological well-being. The World Health Organization defines health as "A state of complete physical, social and mental well-being, and not merely the absence of disease or infirmity[2].” Reflecting this view, the scientific community has placed a stronger emphasis on optimal psychological states in recent years. A number of studies have explored the different types of well-being, suggesting psychological well-being (as opposed to subjective well-being) to be the most comprehensive and most highly correlated to optimal functioning. Although there is growing research on the components and outcomes of well-being, very few studies have focused on interventions that produce optimal functioning or a state of human flourishing. The current study was developed to address this research gap. We suggest one effective method of increasing psychological well-being, the practice of yoga, an age-old practice that has been said to produce physical and psychological health.

## BACKGROUND AND SIGNIFICANCE

Yoga is an ancient system that incorporates mind-body practices, such as breathing, meditation, and physical postures for the purpose of improving physical, emotional, and spiritual well-being. When properly taught, it includes mindful attention on the present experience of the body and mind in order to facilitate deepening levels of awareness. It is said to “prepare the mind for meditation”, by training the attention on gross movements, which is easier and more compelling than the breathing for many people.

While the original purpose of yoga practice was to help the individual develop higher states of consciousness, more recently yoga practice has been utilized as an efficacious adjunct treatment for a variety of psychological and physical disorders. Yoga practice has been shown to improve symptoms of depression, anxiety (for a review, see Khalsa[3]), eating disorders[4], and post-traumatic stress disorder[5]. One study found that yoga practice improved symptoms of stress and anxiety over a simple relaxation training[6].

Research on the efficacy of yoga practice to improve a variety of physical conditions and disorders has also been promising. A 7-week randomized controlled trial of breast cancer patients participating in a yoga intervention reported significant improvements on quality of life, emotional functioning, and a near significant improvement in gastrointestinal symptoms[7]. Studies show that conditions such as asthma, diabetes, obesity, and a variety of musculoskeletal and neurological conditions have also improved due to yoga practice (for a review see Khalsa[3]).

Numerous studies on yoga document significant improvements in mood[8,9,10,11] and stress-coping[10,12], as well as improved performance on cognitive[9,13], psychomotor[9,14], and physical[8,9,15] tasks, suggesting that yoga practice is not only a good intervention for the improvement of disease, but also for the majority of the populations in the healthy range. A cohort study investigating whether or not yoga practice correlated with reduced weight gain in middle-aged women found that those women who had been practicing yoga 4 years or more had a 3.1-lb lower weight gain than nonyoga practitioners[16]. A study investigating the impact of Hatha yoga on physiological and psychological levels in healthy women found significantly higher scores in life satisfaction and lower scores in excitability, aggressiveness, openness, emotionality, and somatic complaints[17]. It is no surprise then that yoga has increased in popularity over the past several decades. A recent survey indicates that 15 million people have tried yoga in their lifetime and that 7.4 million are currently practicing yoga[18]. With these numbers increasing, it becomes ever more important to further scientific understanding of how yoga practice produces benefits in those who practice it.

While many of these studies focus on physical and mental well-being, few focus on behavior change following a yoga teacher training program. Although the varieties of yoga disciplines are abundant (Kundalini, Ashtanga, Hatha), this study focuses on Kripalu yoga, and hints at the importance of coming to a basic understanding of which aspects of yoga practice are the most beneficial. Although many of the studies focus on curing diseases, yoga was originally practiced to develop higher states of awareness, presumably with already relatively healthy individuals. If yoga practice is to reach a larger population, it is important to understand how this practice could benefit physically and emotionally healthy populations.

## METHODS

We evaluated the effects of a 4-week yoga training program housed at the Kripalu Center for Yoga and Health. Kripalu is the country's largest yoga and Ayurvedic training facility. In order to bolster the ecological validity of our study, our preliminary work included three focus groups with yogis and yoginis affiliated with the center to consider what effects should be expected from this yoga program. The resulting constructs were matched with standard validated scales. We also included more traditional measures of psychosocial health (such as optimism), as well as measures designed to capture improvements associated with human flourishing or mindfulness (such as five-factor mindfulness). Our goal was to gather data for a well-powered controlled trial.

The SPSS system[19] was used for all data management and analysis. Outcomes were calculated from paired students' t-tests of difference in means, testing the hypothesis that means were not significantly different per measure over time. Baseline was compared to outcomes after the 4-week training program. The significance of change over a longer term was considered by comparing means at baseline to means at 3 months post-training. Scale items with more than 20% missing data were deleted.

Students entering the program were told about the research study by study staff on site at Kriplau. Interested potential subjects were told more about the study and subjects gave informed consent. A recognized institutional review board provided oversight of the project. Data collection occurred from May 2007 to April 2008 over four yoga teacher training sessions using standardized measurements administered through the SURVEYMONKEY web-based interface[20].

Valid and reliable instruments were used to collect data. Below is listed the measurements used with a brief description. Measurements are grouped as psychosocial or human flourishing scales.

### Psychosocial Measurements

- *Locus of Control (LOC):* The LOC is a 13-item questionnaire that measures the extent to which people believe that they can control the events that affect them. People with a higher internal locus of control believe that their own actions determine the rewards that they obtain, while those with an external locus of control believe that their own behavior does not influence the rewards they receive[21].
- *Perceived Stress Scale (PSS):* The PSS is a brief, validated, and widely used psychological instrument for assessing a subject's perception of stress. The PSS consists of 10 questions that measure the degree to which situations in the subject's life are perceived as stressful, including questions related to perceived unpredictability and lack of control. Participants choose responses ranging from never (0) to very often (4) with a total score ranging from 0 to 40. This scale has become the standard clinical outcome measure for assessing change in stressed individuals[22,23].
- *Self-Efficacy Scale:* The Self-Efficacy Scale is a one-item scale that measures an individual's belief about their ability to perform a given action. In this study, we examined felt belief about maintaining a yoga practice[24].
- *Quality of Life Inventory (QOLI)*: The QOLI has been well-validated on a large population of healthy individuals[25]. It is a 31-item measure that inquires about the satisfaction and importance of different dimensions of a person's life, i.e., relationships, work, creativity. Several longitudinal studies of meditation-based health interventions have demonstrated that quality of life is routinely increased following these interventions[25].
- *The Interpersonal Support Evaluation List (ISEL):* The ISEL measures the perception of social support by measuring across four domains: belonging, self-esteem, appraisal, and tangible help. It is a 14-item scale with good internal validity[26].

- *The Positive and Negative Affect Schedule:* The PANAS consists of 10 positive affects and 10 negative affects in which subjects are asked to rate items on a scale from 1 to 5. It is a well-validated measure that has been correlated to personality traits[27].
- *The Life Orientation Test (LOT):* The LOT was developed to assess individual differences in generalized optimism vs. pessimism. It is a 10-item scale in which subjects are asked to rate items on a scale from 1 to 5. This measure has been used in a good deal of research on the behavioral, affective, and health consequences of this personality variable[28].

### Human Flourishing Measurements

- *Self-Compassion Scale:* The SCS is a 26-item self-report measure of one's ability to be forgiving and kind to oneself in difficult and frustrating circumstances. The SCS has been correlated with positive mental health outcomes, such as less depression and anxiety and greater life satisfaction[29].
- *Five-Factor Mindfulness Questionnaire (5FMQ):* An important goal of Eastern contemplative traditions is the cultivation of a mental factor termed "mindfulness". This mental factor is postulated to encompass several subfactors including attending to internal stimuli, nonjudgment of experience, and acting with awareness. The 5FMQ has recently been developed to measure five of these subfactors. The factor structure of this scale has been validated and collaborators are currently testing its validity in measuring outcome in patients undergoing a meditation-based stress reduction course[30].
- *Self Transcendence Scale:* The Self-Transcendence Scale measures awareness and interest in experiences and information beyond one's own experience[31].
- *Interpersonal Reactivity Index (IRI):* The IRI is a 5-point, 28-item scale that measures empathy. Empathy is measured across four different domains: perspective taking, empathic concern, personal distress, and fantasy. The IRI has good internal validity and consistency[32,33].

## RESULTS

We collected a baseline sample of 46 women and men (85% women). The mean age of subjects in the sample was 41, with an age range of 19–69 years. The sample was self-selected. Most of the sample (92%) self-identified as White, 3% as Black or African American, with 1.5% identifying as Asian and 1.5% reporting that they did not know. The sample was well educated, with most subjects possessing professional training beyond college (47%); 25% graduated from a 4-year college. Nineteen percent had completed part of a 4-year college degree or a 2-year college degree. About half of the sample was employed full time (44%); 12% were employed part time; one subject was a full-time homemaker; 18% were unemployed. The average annual reported income of the sample was high: 44% reported $70,000 or more, 13.4% reported $50,000–65,999, 10.5% reported $35,000–49,999, 14.9% reported $20,001–34,999, and 14.4% reported less than $20,000. The sample reported high levels of health, with only one subject rating her health as less than "excellent". Most reported having no ongoing health problems and more than 80% of the sample did not smoke or drink regularly. The full sample completed the pretreatment forms; 30 subjects completed post-treatment and follow-up measurements.

Table 1 lists the scales used along with mean group differences over time and significance level of any changes over time. The scales are presented as measurements of psychosocial health and measurements of human flourishing. Statistically significant improvements are marked with asterisks. Subjects filled out the entire packet at each of the three data collection points. For significant changes, we offer a brief interpretation of the result.

**TABLE 1**
**Measurements Utilized in Kripalu Yoga Teacher Training Outcomes Project**

| Scale | Baseline to Post-Treatment Mean Difference | Baseline to 3 Months Post-Treatment Mean Difference |
|---|---|---|
| **Psychosocial Health Scales** | | |
| Locus of control: subjects reported significant move to more internal locus of control at 3 months | 0.194(ns) | –0.875* |
| Perceived stress: higher, more stress; subjects felt more stress immediately after program | 4.87*** | 0.92 (ns) |
| Self-efficacy: | 0.07 (ns) | 0.13 (ns) |
| Quality of life | 1.14 (ns) | 1.12 (ns) |
| Perceived social support | 1.00 (ns) | –0.29 (ns) |
| Positive and negative affective states: positive affect factor more positive | –2.94* | –1.12 (ns) |
| Positive and negative affective states: negative affect factor more negative | 1.00 (ns) | 0.75 (ns) |
| Life orientation test: subjects reported significant increases in optimism immediately after program and at 3 months | 1.52** | 2.89**** |
| **Human Flourishing Scales** | | |
| Self-transcendence scale: subjects reported significant increases in transcendence immediately after program and at 3 months | 14.83**** | 9.04** |
| Self-compassion scale | | |
| Total score | 8.90 (ns) | 5.42(ns) |
| Self-compassion scale factors | | |
| Kindness | 2.03 (ns) | 2.21(ns) |
| Judgment | 1.71 (ns) | 0.83(ns) |
| Humanity: subjects reported significant increases in humility at 3 months | 1.52(ns) | 3.92** |
| Isolation | 0.75(ns) | 0.12(ns) |
| Mindfulness: subjects reported significant increases in mindfulness immediately after program and at 3 months | 2.00* | 1.75* |
| Overidentified | 0.87(ns) | 0.75(ns) |
| Five factor mindfulness-total score: subjects reported significant increases in mindfulness immediately after program and at 3 months and at 6 months | –8.16*** | –8.13** |
| Interpersonal reactivity scale factors | | |
| Fantasy | 0.13(ns) | 0.83(ns) |
| Empathy: subjects reported significant increases in empathy immediately after program and at 3 months | –1.77**** | –3.75**** |
| Perspective taking | –0.61(ns) | –1.04(ns) |
| Personal distress: subjects reported significant decreases in transcendence immediately after program and at 3 months | 1.83** | 1.42* |

Significance of differences *$p \leq 0.1$, **$p \leq 0.05$, ***$p \leq 0.01$, ****$p \leq 0.001$, (ns) = nonsignificant.

## DISCUSSION

A goal of this research project was to explore health and higher states of health beyond the absence of illness. Toward this goal, our sample consisted of generally healthy individuals interested in furthering their training in yoga. Given the health of the population and that the training program was designed for personal growth rather than illness treatment, we did not expect to, nor did we find, much change on the psychosocial scales. We included the psychosocial scales to consider possible mechanisms of change, subpopulation analyses, and allow for future explorations in ill, as well as healthy, populations. As expected, in this healthy population, the human flourishing scales showed more change. For example, in this healthy sample, there were no significant changes in perceived social support, quality of life, or self-efficacy from baseline to the 3-month follow-up. However, optimism, a positive psychology research measure, improved from baseline to follow-up.

Perceived stress increased following program participation, most probably due to the transition from intensive yoga training back into home life. Perceived stress returned to baseline at the 3-month follow-up. Similarly, locus of control showed no significant changes postprogram, but became significantly more internal 3 months postprogram, suggesting that positive program effects continue well after the training ends. These findings underscore the importance of longitudinal data collection to understand the full effect of the training and to monitor the persistence of effects. Longitudinal data may be of particular importance in healthy populations as changes may be subtle; a longer observation window allows effects to be observed.

Changes in the human flourishing scales are of particular importance in this study given that yoga theory predicts that training and practice can allow individuals to reach progressively healthier states physically, spiritually, and psychologically. Specifically, the mindfulness subscales of observation, awareness, and nonreactivity all improved following the training, suggesting that one benefit of yoga practice is a more refined ability to attend to one's inner experience. Past studies reveal that increases in mindfulness are associated with personal autonomy, improved connection with one's personal values, and enhanced overall well-being[34].

Although these results are encouraging, there are several limitations to this study. The first is the generalizablility of this sample due to the nature of the yoga training itself. The demographics describe a specific group of individuals participating in this yoga teacher training; white, upper-class women in their 40s. In addition, this is a self-selected population and data were not collected on the individuals who were lost to follow-up, leaving questions as to whether or not these lost individuals continued to receive benefits from the program.

Future work would be strengthened by the addition of selected control groups to better understand which aspects of such a program are effective and for which individuals. The addition of selected biomarkers of health status will increase our mechanistic understanding of the changes found. Still, our initial preliminary investigation into this program is promising, both in terms of feasibility, but also in terms of the ability of the measures to capture changes in this sample. Long-term follow-up data suggest that subjects maintained the positive effects achieved during the program 3 months after the program ended. Thus, the improvements found were lasting into individuals' return to the context of home life.

Knowing how to maximize our ability to stay well can save human suffering and may lower health care costs. The most direct way to investigate this area is to use healthy populations. It is clear that outcomes analysis of programs of healthy individuals require measures sensitive to the types of changes expected, but what are those expected changes? Most health research has focused on an illness model, and health interventions primarily designed for preventing and battling illness. Although there is growing research on the components and outcomes of well-being, very few studies have focused on interventions that produce optimal functioning and human thriving. The current study was developed to address this research gap and found evidence that the practice of yoga improves psychological health. Next steps include narrowing down our measurement package to those scales that best capture changes and considering measurement of additional areas of potential change. Identifying programs that help to maintain health and achieve optimal states of health can help the promotion of practices of empowering self-care and healthier behaviors.
